# Improved tibiofemoral contact restoration after transtibial reinsertion of the anterior root of the lateral meniscus compared to in situ repair: a biomechanical study

**DOI:** 10.1007/s00264-023-05769-y

**Published:** 2023-03-22

**Authors:** Alejandro Espejo-Reina, Maria Prado-Novoa, Alejandro Espejo-Baena, Belen Estebanez, Ana Perez-Blanca

**Affiliations:** 1 Laboratory of Clinical Biomechanics of Andalusia, Escuela de Ingenierías Industriales, Universidad de Málaga, Pedro Ortiz Ramos, s/n., 29071 Málaga, Spain; 2 Clinica Espejo, Málaga, Spain; 3 Hospital Vithas Parque San Antonio, Málaga, Spain; 4 Department of Mechanical Engineering, Universidad de Málaga, Andalucia Tech, Málaga, Spain

**Keywords:** Anterior meniscal root avulsion, Transtibial repair, In situ repair, Biomechanical testing

## Abstract

**Purpose:**

To compare biomechanical behaviour of the anterior root of the lateral meniscus (ARLM) after a transtibial repair (TTR) and after an in situ repair (ISR), discussing the reasons for the efficacy of the more advantageous technique.

**Methods:**

Eight cadaveric human knees were tested at flexion angles from 0° to 90° in four conditions of their ARLM: intact, detached, reinserted using TTR, and reinserted using ISR. Specimens were subjected to 1000 N of compression, and the contact area (CA), mean pressure (MP), and peak pressure (PP) on the tibial cartilage were computed. For the TTR, traction force on the sutures was registered.

**Results:**

ARLM detachment significantly altered contact biomechanics, mainly at shallow flexion. After ISR, differences compared to the healthy group persisted (extension, CA 22% smaller (*p* = 0.012); at 30°, CA 30% smaller (*p* = 0.012), MP 21%, and PP 32% higher (both *p* = 0.017); at 60°, CA 28% smaller (*p* = 0.012), MP 32%, and PP 49% higher (both *p* = 0.025). With TTR, alterations significantly decreased compared to the injured group, with no statistical differences from the intact ones observed, except for CA at extension (15% decrease, *p* = 0.012) and at 30° (12% decrease, *p* = 0.017). The suture tension after TTR, given as mean(SD), was 36.46(11.75)N, 44.32(11.71)N, 40.38(14.93)N, and 43.18(14.89)N for the four tested flexion angles.

**Conclusions:**

Alterations caused by ARLM detachment were partially restored with both ISR and TTR, with TTR showing better results on recovering CA, MP, and PP in the immediate postoperative period. The tensile force was far below the value reported to cause meniscal cut-out in porcine models.

## Introduction

Anterior and posterior meniscal roots are ligamentous structures with direct insertion into the tibial plateau that attach the meniscus to bone, acting as the main restrictors of meniscal extrusion. Focusing on the anterior root of the lateral meniscus (ARLM), its insertion area is located in front of the intercondylar eminence of the tibia beneath the ACL footprint. It forms a complex construct comprising direct bony attachment together with the anterior fibres blended into the anterolateral part of tibial insertion of the ACL [1–3].

Complete detachment of the ARLM may result from a full-width tear within 9 mm of the centre of its tibial insertion or from a soft tissue or bony avulsion. Although little information about the incidence of this lesion is available [3, 4], acute ARLM bony avulsion concomitant with certain tibial fractures [3, 5] and avulsions due to tissue degeneration [6] have been described. Also, in ACL reconstructions, potential iatrogenic damage to the ARLM after reaming the tibial tunnel has been widely recognised [2, 7–10] with reported incidences ranging from 21.7 to 100%, depending on the drilling method and the diameter of the reamer [10], and cases of root disengagements due to this cause have been informed [11, 12]. For these reasons, probing the ARLM root after tunnel drilling has been specifically recommended to guarantee its attachment, or, otherwise, meniscal extrusion [2] and later osteoarthritis could be iatrogenically instigated. The clinical consequences of ARLM disinsertion can be serious, since it disrupts the circumferential fibres of the meniscus, which can severely compromise meniscal function. Complete anterior root tears have been associated with tibial cartilage and meniscal deterioration in animal models [13–16]. A recent work [16] found that detachment of the ARLM generated alterations of knee contact biomechanics of a similar magnitude to posterior root detachment, but producing the highest effects at shallow flexion angles [17] instead of in deep flexion as observed with the latter [18]. Given that daily life involves longer periods of knee loading at low flexion, preservation of ARLM integrity may be of even greater clinical significance than with posterior root tears [19, 20].

Once disinsertion has been confirmed, surgical root repair reconnecting the meniscus to bone in its anatomical position aims to restore joint kinematics, contact pressures, and delay the development of OA [19]. The clinical [20] and biomechanical [18, 21–25] success of surgical repair of posterior meniscal roots has been studied previously. To repair the anterior roots, several authors have conducted in situ repair (ISR) on the medial [26] and lateral meniscus [6, 17] because of the easiness of the technique in these anatomical locations. In a biomechanical study, ISR has shown a partial recovery of the contact alterations induced by an ARLM avulsion [17], outlining the benefits of the surgical intervention but also indicating room for improvement in the outcomes. In this line, an alternative to ISR is the application of a transtibial repair (TTR) technique, recognised as the gold standard for posterior root detachments of the medial meniscus [27] and also applied to the posterior root of the lateral meniscus [18] and the anterior root of the medial meniscus [28]. The question arises as to whether TTR may be a better treatment option than ISR to repair an avulsed ARLM, as there is a lack of information on the subject.

Therefore, to add data that may assist the surgeon in deciding which surgical technique to apply, the aim of this work has been to evaluate the success of TTR compared to ISR in restoring pre-injury knee contact biomechanics after ARLM avulsion, in a simulated immediate postoperative period. Additionally, the role of suture tension in the success of the repair has been analysed.

## Materials and methods

After approval by the ethical committee of our university, eight cadaveric knees with no signs of pathologies were included in the study (five men and three women; ranging between 68 and 91 years of age). The specimens were provided by a specialised company that took care of the subsequent incineration. The same expert surgeon performed all preparations and surgical simulations.

### Specimen preparation

**Fig. 1:**
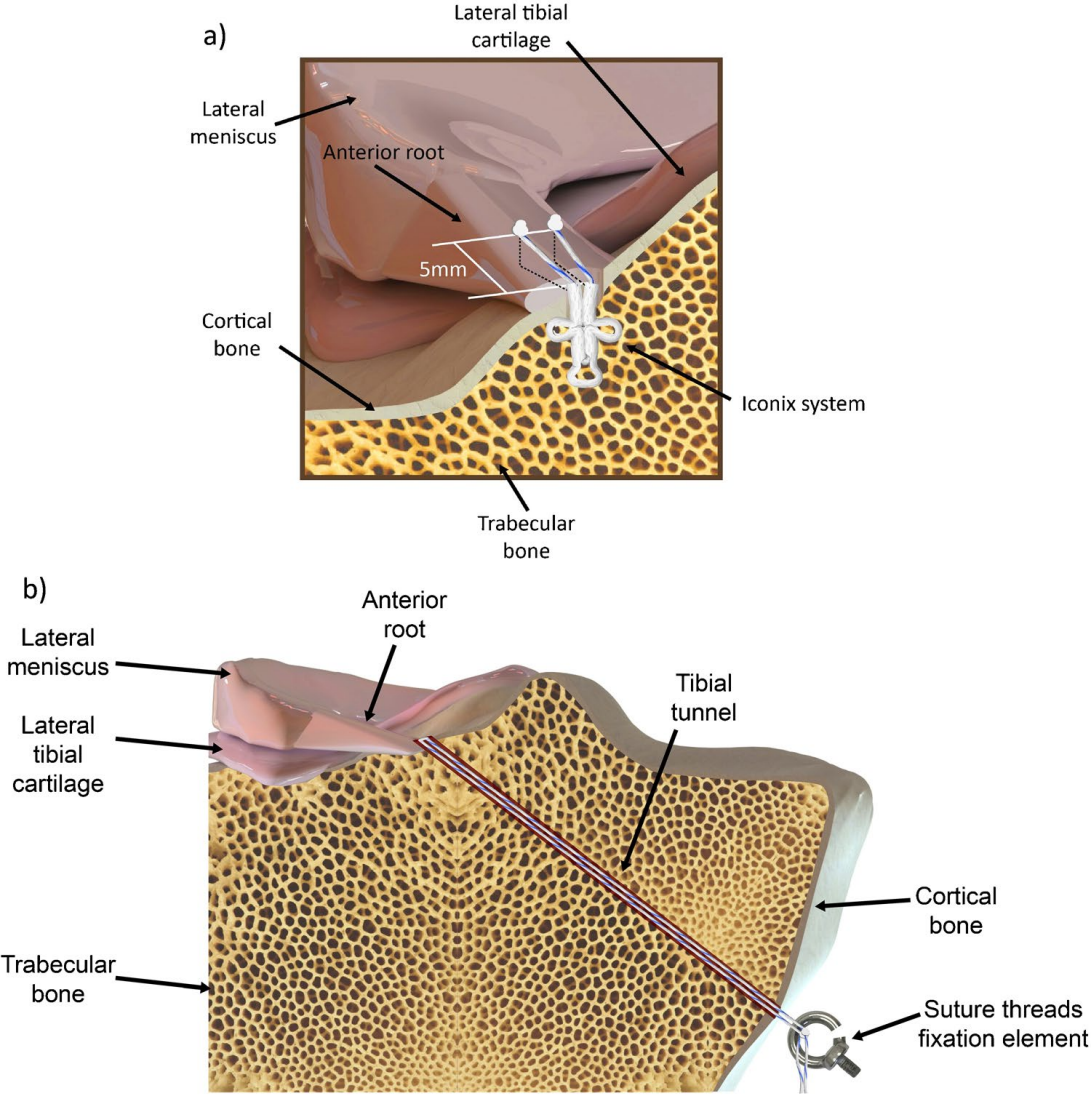
Schematics of ARLM repairs: **a** ISR technique, **b** TTR technique

**Fig. 2:**
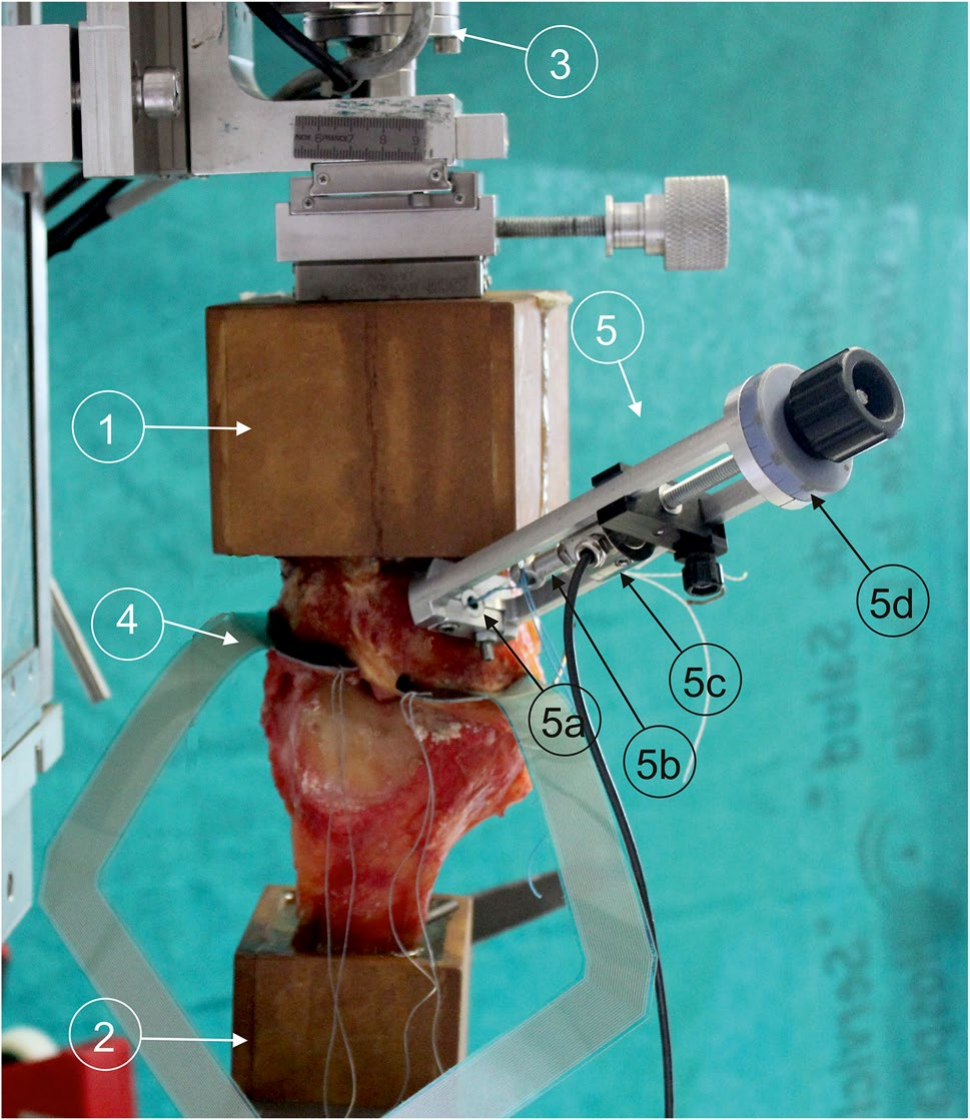
Experimental setup showing the testing machine with a left knee specimen mounted at full extension in the TTR condition. (1) Tibial container; (2) femoral container; (3) load cell; (4) pressure sensor; (5) custom-designed transducer, showing (5a) screw that fixes the transducer to the tibial tunnel, (5b) element to fix the suture threads, and (5c) load cell; (5d) calibrated wheel that displaces the thread fixation element in the direction of the tibial tunnel

Femora and tibiae were cut to 120 mm and 150 mm from the articular surface, respectively, and the specimens were prepared according to Perez-Blanca et al. [18]. They were tested in four lateral meniscus conditions: (1st) intact; (2nd) injury; (3rd) ISR; (4th) TTR. After completion of the test for the intact condition, the injury condition was simulated and tested. The ARLM was detached by completely sectioning its circumferential fibres with a scalpel at approximately 5 mm from the centre of its tibial insertion, including those braided with the ACL. For the ISR (Fig. 1a), a suture anchor (Iconix® 2.3 mm, two threads; Stryker, Greenwood Village, CO, USA) was inserted at the ARLM anatomical footprint and its threads pulled until a hard stop was felt. One limb of the first thread was passed through the meniscus in the junction between the peripheral and the intermediate meniscal thirds and one limb of the second thread in the junction between the intermediate and the inner meniscus thirds. Finally, each limb was sutured to its corresponding free limb, embracing the meniscus on its peripheral and on its inner side, respectively. As a representative measure of the free length of thread in ISR, with the knee unloaded, the distance between the entry point of the suture anchor on the tibial surface and the midpoint between suture holes on the proximal surface of the meniscal horn was measured using a calliper. After the third condition test was finished, the suture anchor was removed and the TTR performed (Fig. 1b). A 3.2-mm tibial tunnel was drilled with entry at ARLM insertion overlapping the ACL footprint 1 mm laterally, taking care not to further harm it, and with exit 1 cm anterior to the anterior edge of the medial collateral ligament and 1 cm proximal to the hamstring tendon. As a representative magnitude of the free length of thread in TTR, the tunnel length was measured between the approximate centre points of tunnel exits using a digital calliper. Then, a no. 2 suture thread (Force Fiber, Stryker, Endoscopy, San José, CA) was inserted through each hole, previously created in the meniscus for the ISR, and both limbs of each suture passed through the tibial tunnel. The threads were tied to a specifically designed transducer (Fig. 2), capable of recording the traction force with a class 0.2 load cell of 200 N rating (HBM, Darmstadt, Germany) and of controlling the suture elongation in the tunnel direction. The transducer was firmly fixed to the distal exit of the tibial tunnel.

### Biomechanical testing

The knees were tested in a universal testing machine [29] (Fig. 2), controlling the flexion angle while preserving the other degrees of freedom [18]. A class 1 load cell of 2kN rating (HBM, Darmstadt, Germany) measured the load. Piezoresistive mapping sensors (K-scan 4000, Tekscan Inc., Boston, MA) were introduced between the meniscus and the tibia to register cartilage pressures (Fig. 2), using a new sensor for each specimen. Immediately prior to use, each sensor was preconditioned with five cycles of 1000 N, and subsequently a three point grade calibration was performed, following the manufacturer’s protocol. The tips of each of the two transducer sensing zones were reinforced with adhesive tape; then Dyneema® threads were passed through them and used to guide the insertion of the sensor from anterior to posterior. The coronary ligament of the specimen was sectioned only as necessary to insert the sensor between each meniscus and the tibia [17, 22], taking care not to damage the menisci and ligaments of the knee. Since the sensitive areas of the sensor do not exactly match the intra-articular surfaces, they were positioned to maximise coverage of the contact zones for each test condition under manual knee compression, as observed in the raw real-time sensor results. Once both sensor areas were in place, the Dyneema® threads were tied to screws attached to the tibial container to minimise sensor movement during testing.

For each meniscal condition, the specimen was tested at four flexion angles sequentially: 0°, 30°, 60°, and 90°. A 100 N compression was slowly applied while permitting natural knee realignment. Then, the anteroposterior displacement was blocked, to simulate the stabilising action of the muscles [30, 31]. For the TTR group, at this point, the suture tension was reset at 15 N. A fixed initial tension value was chosen to discard possible loosening caused by previous tests, which could be important in this group because of the length of the thread. Finally, the compression load was increased to 1000 N, and pressures on the tibial cartilage were recorded after one min under maximum compression to allow stabilisation of the sensors [32, 33]. Load level and flexion range were selected based on previous studies [17, 18, 24, 34, 35] to facilitate comparisons although values are greater than those expected in the postoperative period.

From the pressure sensors, contact area (CA), mean pressure (MP), and peak pressure (PP) at each tibial compartment were computed, excluding pressures below 0.07 MPa [33]. In the TTR group, the maximum suture tension during knee loading and after one min at 1000 N were identified. All experimental data were processed using Matlab® v. R2019b (MathWorks Inc., Massachusetts, USA).

### Statistical analyses

CA, MP, and PP were normalised according to their values with intact menisci for the same specimen, condyle, and flexion angle to control inter-specimen variability. Suture traction forces at different flexion angles were normalised with respect to the homologous instant for the same knee in extension.

Nonparametric tests were applied to assess for differences between testing conditions. The normalised CA, MP, and PP for each condition were compared with 1 to assess any variation relative to the intact knee using the Wilcoxon signed-rank test. Friedman’s analysis of the variance test was applied to evaluate differences between conditions. When an overall significant difference was detected, a Wilcoxon signed-rank test with a Bonferroni correction was performed for pairwise comparisons. SPSS Statistics® v. 25 (IBM, Chicago, IL, USA) was used for all analyses; *p* ≤ 0.05 was considered statistically significant. For suture tractions in TTR, differences at each flexion angle relative to extension were analysed comparing the normalised values with 1 using the Wilcoxon signed-rank test.

Since clinically relevant differences in contact parameters are unknown, the normalised PP at the lateral compartment of the first three specimens was used to select the group size. A minimum size *n* = 7 was obtained with the Wilcoxon signed-rank tests for a computed effect size of 1.5 between the injured and intact conditions and *n* = 7 with the Friedman test for an effect size of 2.19 between treatment conditions using *α* = 0.05 and a power of 0.8. A conservative sample size *n* = 8 was chosen, which is in accordance with prior studies [18, 21, 22, 24, 35, 36].

## Results

The representative free lengths of thread were 33.1(SD 3.8)mm for the TTR group and 5.3(SD 0.5)mm for the ISR group. The outcomes related to the tibiofemoral contact analysis (CA, MP, and PP) for the 4 conditions tested are reported below, along with the statistical comparisons between conditions. In the last subsection, the results of the traction force on the suture of the TTR group are presented. All data are normalised as described in the “Statistical analyses” section.

### Normalised CA (Table 1, Fig. 3a,d)

**Table 1:** Normalised contact parameters at the lateral compartment at each flexion angle for the 3 altered meniscal conditions (given as mean value with 95% confidence interval in parentheses). p value next to the corresponding symbol when detecting a significant difference

		*0*	*30°*	*60°*	*90°*
CA	Injury	0.55 (0.08) * *p* = 0.012	0.61 (0.11) * *p* = 0.012	0.60 (0.15) * *p* = 0.017	0.76 (0.15) * *p* = 0.025
	ISR	0.78 (0.05) * *p* = 0.012 ◊ *p* = 0.024	0.70 (0.09) * *p* = 0.012	0.72 (0.14) * *p* = 0.012 ◊ *p* = 0.025	0.81 (0.18)
	TTR	0.85 (0.07) * *p* = 0.012 ◊ *p* = 0.024	0.88 (0.08) * *p* = 0.017 ◊ *p* = 0.034	0.94 (0.09) ◊ *p* = 0.034	0.99 (0.20)
MP	Injury	2.08 (0.45) * *p* = 0.018	2.13 (0.39) * *p* = 0.012	1.85 (0.42) * *p* = 0.012	1.35 (0.24) * *p* = 0.036
	ISR	1.02 (0.20) ◊ *p* = 0.036	1.21 (0.26) * *p* = 0.017 ◊ *p* = 0.034	1.24 (0.20) * *p* = 0.025 ◊ *p* = 0.024	0.90 (0.22) ◊ *p* = 0.036
	TTR	0.92 (0.17) ◊ *p* = 0.036	0.88 (0.18) ◊ *p* = 0.024	0.82 (0.18) ◊ *p* 0.024	0.62 (0.31)
PP	Injury	2.41 (0.54) * *p* = 0.018	2.53 (0.55) * *p* = 0.017	1.99 (0.69) * *p* = 0.012	1.42 (0.45)
	ISR	1.19 (0.44) ◊ *p* = 0.036	1.32 (0.33) * *p* = 0.017 ◊ *p* = 0.05	1.49 (0.41) * *p* = 0.025	1.00 (0.38)
	TTR	1.07 (0.32) ◊ *p* = 0.036	1.01 (0.31) ◊ *p* = 0.05	0.92 (0.32) ◊ *p* = 0.034	0.85 (0.38)

*represents the significant difference with respect to the intact condition; ◊ represents the significant difference with respect to the injured condition

**Fig. 3:**
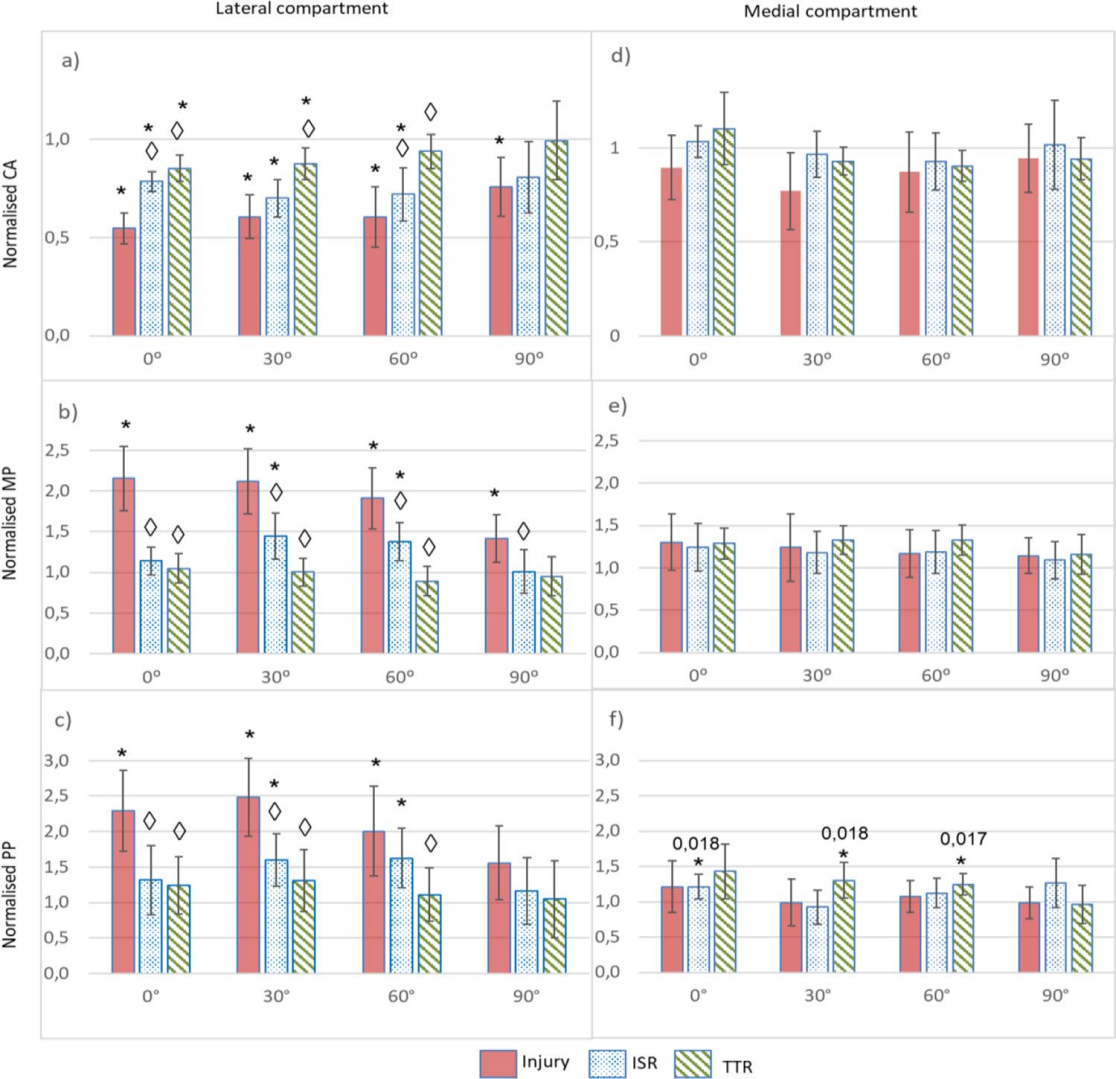
Mean values of the normalised biomechanical parameters for the three altered meniscal conditions at the four knee flexion angles tested, with the vertical line representing the 95% CI interval: **a** normalised CA, **b** normalised MP, and **c** normalised PP at the lateral compartment and **d** normalised CA, **e** normalised MP, and **f** normalised PP at the medial compartment. * represents the significant difference with respect to the intact condition. ◊,represents the significant difference with respect to the injured condition. For the results in the medial compartment, the *p* value is indicated when detecting a significant difference

At the lateral compartment, the lesion significantly changed the CA for all flexion angles tested. When repairing, it was observed that the normalised mean value was always closer to 1 (the intact condition) for the TTR group than for the ISR group. Analysing in detail both repairs, by using an in situ technique, the improvement with respect to the injury group was significant at extension and at 60°; however, significant differences from the intact condition remained except at 90°. When repairing with transtibial technique, improvement from the injury group was significant at all angles except at 90°, and with differences from the intact condition only at extension and at 30°.

At the medial condyle, no significant differences were found.

### Normalised MP (Table 1, Fig. 3b,e)

At the lateral compartment, the MP was significantly increased by the lesion at all flexion angles tested. After ISR, significant improvements with respect to the injury group were observed at all angles; however, there was still a significant increase compared to the healthy group at 30° and at 60°. After TTR, also significant improvements from the injury group were achieved except at the maximum angle tested, 90°, where just a tendency to difference was achieved (*p* = 0.056) probably due to the large dispersion in this position. It is noteworthy that differences from the intact condition disappeared for all positions with TTR.

At the medial condyle, no difference was found.

### Normalised PP (Table 1, Fig. 3c,f)

At the lateral condyle, a significant increase in the PP caused by the injury was also observed except at 90°. The success in decreasing such rise was superior with the TTR for all the positions in which significant differences from the injured group were detected. Specifically, after ISR, significant improvement from the injury group could only be detected in extension and at 30°, while the increase in PP with respect to the intact condition continued to be detected at 30° and 60°. By comparison, significant improvement with respect to the injury group was observed after TTR at all positions tested except at 90°, while no differences from the healthy group were maintained.

At the medial condyle, a significant increase from the intact condition was observed with ISR at extension and with TTR at 30° and at 60°; no other differences were detected.

### Thread traction force (Table 2)

Data from two specimens at 90° and one specimen at 60° were discarded as outliers.

A clear increase in the suture traction force was always observed as the compressive load on the knee rose. By pooling the percentage of increase across all flexion angles, an average maximum increase of 398% (SD 0.91) at 1000 N of compression was observed. Subsequently, a relaxation phenomenon which led to a mean increase of 322% (SD 0.83) after the first minute was observed.

When normalising the traction force after 1 min of maximum compression according to its value for the knee in extension, a significant difference was observed only at 30° with a mean increase of 26% (*p* = 0.015).

**Table 2:** Suture traction force for the TTR group after 1 min at 1000 N of compression (given as mean value with 95% confidence interval in parentheses)

	0	30°	60°	90°
*Tension force (N)*	36.46 (11.75)	44.32 (11.71)	40.38 (14.93)	43.18 (14.89)
*Normalised tension force*	1	1.26 (0.17) *	1.09 (0.08)	1.29 (0.27)

*represenys the significant difference with respect to traction force in full extension

## Discussion

The results showed that while the biomechanical alterations caused by ARLM avulsion were partially restored by both ISR and TTR in terms of recovery of the CA, MP, and PP, the recovery was more successful using TTR.

Specifically, TTR achieved a significant improvement by more than 50% compared to the injured group for all the studied parameters at extension, at 30° and 60°. Furthermore, the dispersion being of the same order for the injured as for the repaired groups, TTR succeeded in eliminating statistical differences from the healthy group for pressure-related parameters although a slight decrease of the CA persisted at extension and at 30°. ISR also attenuated the effects of the avulsion but to a lesser extent: differences from the healthy group were observed for the pressure-related parameters, except in extension; compared to the healthy group, the CA was considerably smaller after ISR than after TTR in extension (reduction by 22% vs. 15%), at 30° (reduction by 30% vs. 12%) and at 60° (reduction by 28% vs. 6%). Regarding the 90° flexion, no significant differences with respect to the healthy group were found for any repairing technique, except for the MP after ISR, but none from the injury group, probably due to the lesser influence of the avulsion at this position.

We believe that the role of the stiffness of the fixation can be important, since the higher the stiffness, the lower the root displacement for the same force stimuli. Suture stiffness is particularly important for TTR, because of the much longer threads. It could be hypothesised that a reason for superior results in TTR was that the stiffness of the root repaired with this technique was closer to its intact value. Conversely, the lower elongation achievable by the suture of the ISR would limit the mobility of the meniscus necessary to adapt to the variable location of the CA, worsening the results. If further research confirms this hypothesis, it would not be true that the stiffer the suture is, the better the results at the immediate postoperative period are and the best mechanical properties for surgical sutures subjected to physiological loading conditions [37] should be studied for each repair technique with its characteristic suture length.

Maximum suture forces measured in TTR were more than four times lower than those reported to initiate the meniscus cut-out in a porcine model [38], reported as the main cause of permanent displacements in the postoperative period [39]. The large difference between suture force and the aforementioned value leads us to believe that the risk of this postoperative complication is low. Regarding ISR, although higher traction force would be needed to allow similar meniscus mobility due to shorter threads, it was not measured in this group, and more variables influence on meniscal root stress. The force generated at the ARLM to restrain its displacement induced by axial compression was significantly higher, compared to its value at extension, only at 30°, but not at 60° and 90°. This finding reinforces that root avulsion and its repair are especially determinant at low flexion angles, as we observed in the contact parameters and as it was previously reported [17].

The anatomical coupling of the tibial footprints of ACL and ARLM, with reported overlaps of 63.2% of the ARLM and 40.7% of the ACL tibial footprints [2, 3], is probably the reason for the high risk of ARLM iatrogenic lesions in ACL surgical procedures [2, 8–11]. It is expected that most of these iatrogenic injuries produce partial and not complete detachment of the ARLM, but it could progress postoperatively [11]. Besides, slight deviations of the ACL tunnel during drilling are not rare and may cause a greater damage [8, 10] that may be difficult to detect [9], so careful inspection of meniscal stability is recommended. It is important to mention that, while the influence of the position of tibial tunnel aperture for ACL reconstruction on the attachment of the lateral meniscus has been studied in terms of meniscal extrusion [2], we are not aware of any study analysing the influence of the position of the tunnel for TTR of the ARLM on ACL integrity, so further investigation is warranted. Furthermore, special care should be taken when both lesions need treatment, as the tunnels required for their possible reconstruction may converge.

Various surgical techniques have been proposed to treat meniscal root avulsions; however, most biomechanical studies on the assessment of the repairs focus on the medial meniscus and mainly on its posterior root [21–23], establishing the transtibial technique as the gold standard [27]. For the lateral meniscus, application of TTR techniques to reattach the posterior root has been reported, and some studies evaluated the success of the repair [18, 40]. Regarding avulsions of the anterior roots, ISR techniques have been proposed for both lateral [6] and medial meniscus [26], because of the easiness of application in this anatomical location. Also, TTR techniques have been applied to repair both anterior roots of the medial [28] and lateral meniscus [3].

We do not know any previous study evaluating the success of the TTR of the ARLM, and even less comparing its efficacy against the use of ISR. We only know a recent study [17] that has biomechanically evaluated the surgical repair of the ARLM applying an ISR technique. Using cadaveric knees subjected to 1000 N compression at flexion angles from 0 to 90°, it was found that CA, MP, and PP partially recovered towards the levels of the intact condition after repairing, mainly at low flexion angles where the injury had produced the higher alterations. However, statistically, differences from the intact condition were still observed, showing that the recovery was incomplete. Prince et al. [41] studied a two cm longitudinal tear in the peripheral third of the anterior horn of the lateral meniscus, although the simulated injury did not fully disrupt meniscal fibres as root avulsion does. They found no alterations in contact biomechanics caused by neither the tear nor its repair mimicking an all-inside technique with bony fixation to the anterior tibia with two separate knotless anchors. When a partial meniscectomy at the ARLM area was simulated, involving fully disruption of the meniscal fibres, a significant increase in the PP and CA in the injured compartment with respect to the intact knee was found.

## Limitations

Only the immediate postoperative period could be simulated since the biological response of tissues was not reproduced. Therefore, the influence of meniscus mobility on the healing process was not assessed. No muscle activity was reproduced, which required blocking anteroposterior displacement to achieve knee stability. This is common practice [17, 30, 42], but we are aware that the natural alignment of the specimen may be altered [30]. To lessen possible alteration, a procedure that kept all degrees of freedom of the knee, except controlled flexion, until reaching 100 N of compression was adopted, and only then the anteroposterior displacement was disabled. Special care was taken to minimise anatomical alterations of the joint, but a considerable amount of soft tissue from the anterior knee aspect had to be removed, and the coronal ligament had to be partially sectioned to allow insertion of the sensors, which is also a habitual procedure [17, 18, 21–23, 30, 41]. To limit the influence on the results of the possible diminishing of the load output measured by Tekscan sensors [33], the pressure in each test was normalised by the total applied force.

The number of specimens in each group is small; a larger number of specimens would provide more solid conclusions especially in those positions with higher statistical dispersion. However, as mentioned in the “Statistical analyses” section, a minimum group size calculation was performed in trying to ensure sufficient power to support our conclusions. On the other hand, such sample size is in line with previous studies [18, 22, 24, 35, 36, 40].

## Conclusions

Alterations caused by ARLM detachment were partially restored with both ISR and TTR, with TTR showing better results on recovering CA, MP, and PP in the immediate postoperative period. The tensile force was far below the value reported to cause meniscal cut-out in porcine models.
